# Chemogenomic screening identifies the Hsp70 co-chaperone DNAJA1 as a hub for anticancer drug resistance

**DOI:** 10.1038/s41598-020-70764-x

**Published:** 2020-08-14

**Authors:** Nitika, Jacob S. Blackman, Laura E. Knighton, Jade E. Takakuwa, Stuart K. Calderwood, Andrew W. Truman

**Affiliations:** 1 Department of Biological Sciences, University of North Carolina Charlotte, Charlotte, NC 28223 USA; 2 Department of Radiation Oncology, Beth Israel Deaconess Medical Center, Harvard Medical School, 330 Brookline Ave, Boston, MA 02215 USA

**Keywords:** Chaperones, Chemical genetics

## Abstract

Heat shock protein 70 (Hsp70) is an important molecular chaperone that regulates oncoprotein stability and tumorigenesis. However, attempts to develop anti-chaperone drugs targeting molecules such as Hsp70 have been hampered by toxicity issues. Hsp70 is regulated by a suite of co-chaperone molecules that bring “clients” to the primary chaperone for efficient folding. Rather than targeting Hsp70 itself, here we have examined the feasibility of inhibiting the Hsp70 co-chaperone DNAJA1 as a novel anticancer strategy. We found DNAJA1 to be upregulated in a variety of cancers, suggesting a role in malignancy. To confirm this role, we screened the NIH Approved Oncology collection for chemical-genetic interactions with loss of DNAJA1 in cancer. 41 compounds showed strong synergy with DNAJA1 loss, whereas 18 dramatically lost potency. Several hits were validated using a DNAJA1 inhibitor (116-9e) in castration-resistant prostate cancer cell (CRPC) and spheroid models. Taken together, these results confirm that DNAJA1 is a hub for anticancer drug resistance and that DNAJA1 inhibition is a potent strategy to sensitize cancer cells to current and future therapeutics. The large change in drug efficacy linked to DNAJA1 suggests a personalized medicine approach where tumor DNAJA1 status may be used to optimize therapeutic strategy.

## Introduction

Hsp70 is a molecular chaperone that plays important roles in protein quality control processes such as protein folding, transport, degradation, and the prevention of protein aggregation^1^. Hsp70 levels are elevated in various cancers and overexpression correlates with poor prognosis for survival and response to cancer therapy^2^. The elevated levels of Hsp90 and Hsp70 chaperones in cancer and their role in fostering multiple oncogenic pathways has made these proteins attractive drug targets with numerous anti-chaperone compounds having been developed so far^3^. Problematically, Hsp70 is required for cell survival and protein homeostasis, and thus its inhibition is detrimental to the viability of both normal and cancer cells, with dubious selectivity for tumor cells^4^.

Hsp70 performs all its functions in association with a large spectrum of helper proteins known as co-chaperones that include J-proteins, tetratricopeptide repeat (TPR) domain-containing proteins and nucleotide exchange factors (NEFs) which fine-tune Hsp70 specificity and activity in the cell. The J-proteins recruit the protein substrates or clients and interact with such clients at the interface of NBD and SBDβ of Hsp70. This interaction leads to increased Hsp70-mediated ATP turnover and activation of protein folding. J-proteins have a highly conserved 70 amino acid motif containing Histidine, Proline and Aspartic acid amino acid residues known as HPD motif which is essential for stimulating ATPase activity of Hsp70^5^. In humans, the J-protein family has about 50 members which are further divided into three groups based on the localization of J-domain within a protein^6^. The Hsp40 DNAJA1 (more commonly referred to as DNAJA1) associates with unfolded polypeptide chains, preventing their aggregation^6^. Several Hsp70 inhibitors have failed in clinical trials due to their toxicity. More recently, alternative strategies have focused on sensitizing cells to anticancer agents by either manipulating post-translational modification of chaperones or their interaction with specific co-chaperones^4,7–11^.

DNAJA1 (mammalian homolog of yeast Ydj1) is an interesting possible anticancer target as a key mediator of Hsp70 function that appears to regulate specific features of tumorigenesis^8,12^. A recent study demonstrated that CRPCs expressing ARv7 are insensitive to Hsp90 inhibitors but are sensitive to Hsp40 inhibition^13^. In addition, we have shown that targeting specific oncoprotein complexes (ribonucleotide reductase) with a combination of traditional as well as a DNAJA1 inhibitor produces highly synergistic effects^8^. We propose that targeting DNAJA1 in cancer may offer an attractive alternative to the toxicity induced by full Hsp90/Hsp70 inhibition.

Anticancer monotherapies using broadly active cytotoxic or molecularly targeted drugs are limited in their ability to demonstrate a reliable clinical response. This is due to redundant signaling pathways, feedback loops and resistance mechanisms in cancer cells^14^. Thus, combination anticancer therapies have been used clinically for over 50 years to improve the responses achieved by monotherapies alone. Cancer cell line-based models for these combination therapies are easy and inexpensive to perform using high-throughput drug screening protocols (HTS) to identify the most effective drug combination^15,16^. HTS helps to explore the relationship between the cell line characteristics and drug specific dose responses^15^. Chemogenomics is one such HTS-based approach where a large collection of anticancer chemical drugs are screened to identify biological targets. These screening sets often contain small molecules that are well annotated and have defined molecular targets. Such an approach is particularly beneficial for cancer research because malignant cells often contain multiple aberrations that require targeted therapy to inactivate cancer driver activities and mitigate deleterious effects of the drugs to normal cells^14^.

Here, we performed an unbiased screen of the NIH Approved Oncology Drug set containing 131 anti-cancer drugs in combination with HAP1 cancer cell lines depleted of J-protein DNAJA1. We identified 41 compounds showing strong synergy with the loss of DNAJA1, and in contrast 18 molecules that displayed reduced potency in the knockout cell line. We validated three drugs (cabozantinib, clofarabine and vinblastine) in combination with a unique DNAJA1 inhibitor (116-9e) for synergy in the LNCaP cancer cell lines and confirmed omacetaxine mepesuccinate, idarubicin and sorafenib for antagonism (i.e. with reduced potency after DNAJA1 inhibition). This study demonstrates the validity of developing Hsp70 co-chaperone inhibitors to sensitize cells to current anticancer therapies and suggests that determining DNAJA1 status of a tumor may be beneficial in selecting the most appropriate course of treatment.

## Results

### *DNAJA1* is mutated and overexpressed in a variety of cancers

**Figure 1:**
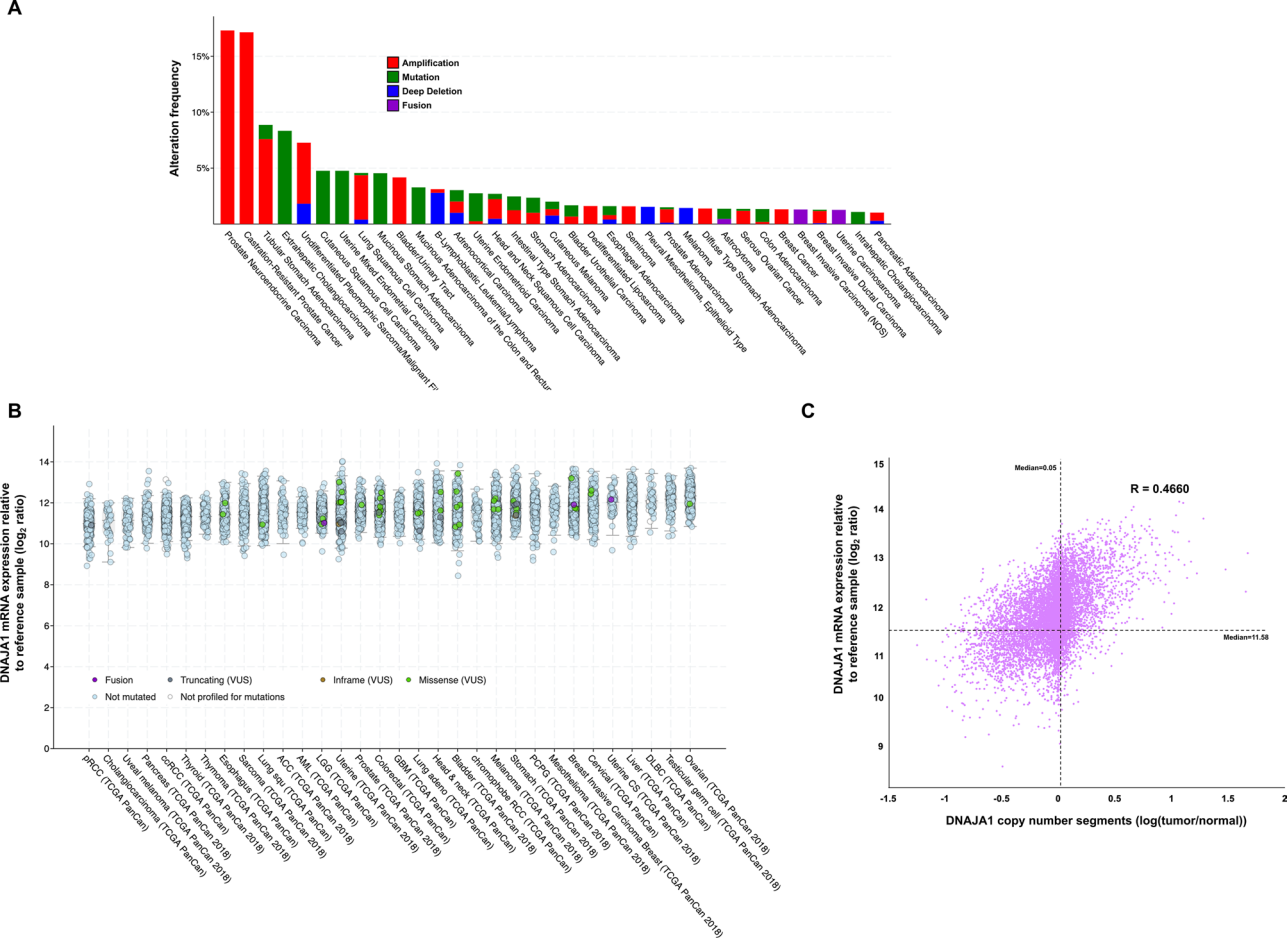
*DNAJA1* is altered in cancer. (**A**) Prevalence of *DNAJA1* alterations in various cancer genomes analyzed via the cBioPortal. (**B**) *DNAJA1* mRNA expression in cancers (TGCA PanCan) obtained from cBioPortal. mRNA expression value is log2 ratio of expression seen in cancer vs. reference cells (please see www.cbioportal.org/faq for more information). (**C**) increased *DNAJA1* expression is not driven by copy number increase. *DNAJA1* copy number vs *DNAJA1* expression was plotted and Pearson’s correlation coefficient (R-value) was calculated. Median of both variables is marked by dotted line on the graph.

While the roles of Hsp90 and Hsp70 in cancer have been thoroughly studied, much less is known of the role that regulatory co-chaperone proteins such as DNAJA1 play in tumorigenesis. As a first step, we queried the cBioPortal cancer genomic database (cbioportal.org) to determine the incidence of *DNAJA1* alterations in cancer. Analysis of data from 176 non-redundant studies representing 44,347 patient samples revealed that *DNAJA1* was altered at a frequency of greater than 1% in 35 cancer types (Fig. 1A). Although the majority of alterations in *DNAJA1* occur at a relatively low frequency (< 5% of cancers) *DNAJA1* is significantly amplified in prostate neuroendocrine cancer (PNC) and castration-resistant prostate cancer at a frequency of 17.31% and 17.14% respectively (Fig. 1A). Hsp70 and Hsp90 are often overexpressed in tumors^2^. To determine whether the *DNAJA1* expression is also overexpressed in cancer, we analyzed *DNAJA1* mRNA expression in samples from the TGCA PAN-CAN Atlas. Interestingly, *DNAJA1* mRNA was expressed at significantly higher levels in these samples, with a median expression in cancer over 3,000 × relative to WT reference samples (Fig. 1B). To determine if this dramatic overexpression of *DNAJA1* was a result of amplification, we plotted *DNAJA1* expression vs amplification (Fig. 1C). Interestingly, there was minimal correlation between amount of amplification and *DNAJA1* expression (r = 0.45) suggesting that while *DNAJA1* may be an important marker in cancer it is not caused by gene amplification.

### Characterizing the role of DNAJA1 in anticancer drug resistance

**Figure 2:**
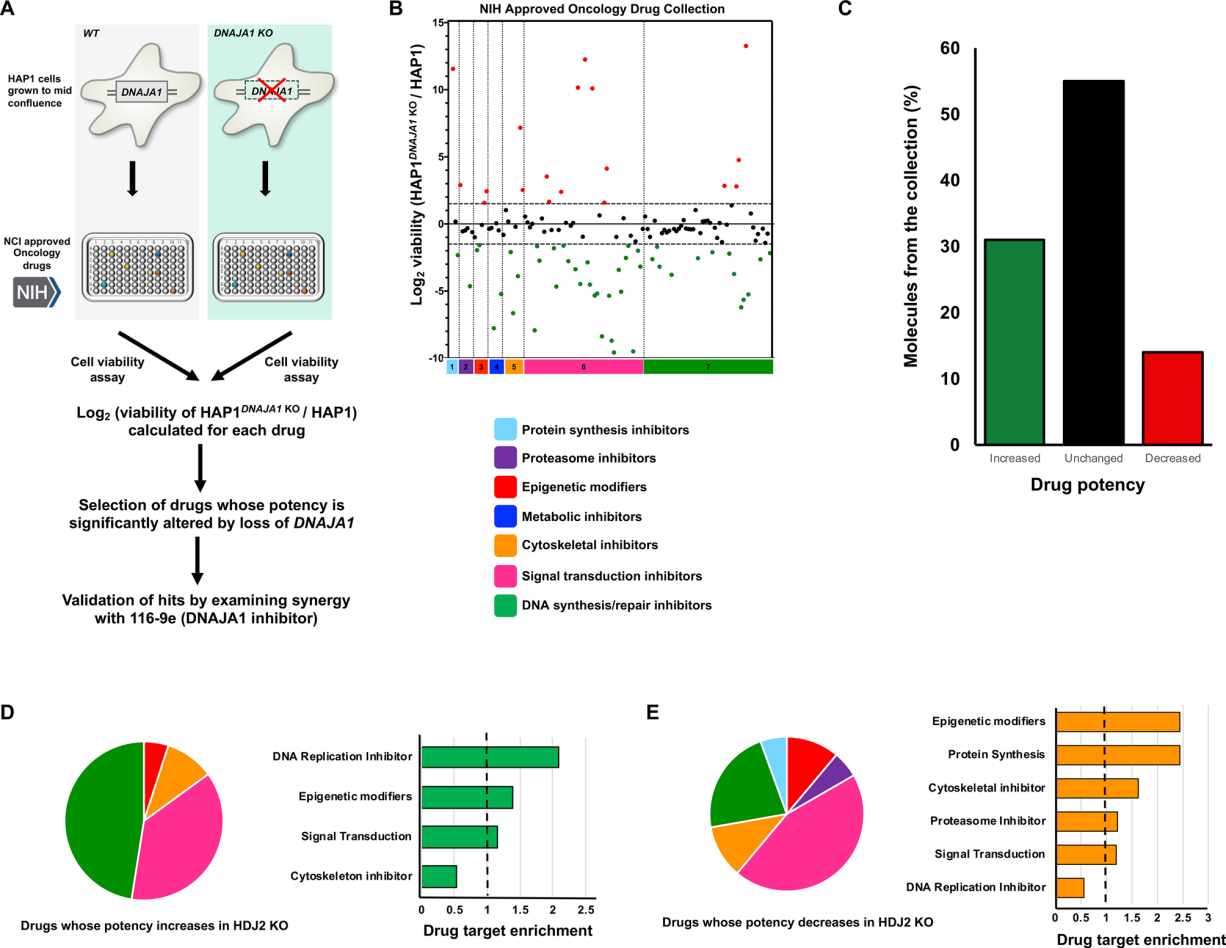
Sensitivity of WT and *DNAJA1* knockout cells to the NIH Approved Oncology Collection. (**A**) Workflow of high-throughput cell-based screen. (**B**) A collection of 132 drugs were screened at 50 μmol/L with Wild-type and *DNAJA1* KO cells. Results are the average of at least triplicates and error is SEM. The dotted lines represent a potency change of Log_2_ > 1.5 or Log_2 _<  − 1.5. The effect of *DNAJA1* knockout on drug potency is colored as follows: red (decreased drug potency), green (increased drug potency) or black (no change in drug potency). (**C**) Summary of effect of *DNAJA1* knockout on the potency of the NIH approved oncology collection. (**D, E**) Drug ontology of synergistic and antagonistic hits based on the pathways affected by the approved oncology drugs in the screen.

The existing literature is contradictory as to whether DNAJA1 may possess tumor suppressor or driver properties^12,17^. To clarify whether silencing of *DNAJA1* could be beneficial in the treatment of cancer, we screened wildtype HAP1 cells and HAP1 cells lacking *DNAJA1* (HAP1^DNAJA1 KO^) for comparative resistance against the NIH NCI Approved Oncology Collection (Fig. 2A) (https://dtp.cancer.gov/organization/dscb/obtaining/available_plates.html). Prior to screening, we validated the status of the *DNAJA1* knockout cell line by Western blotting for DNAJA1 and other major chaperones and co-chaperones (Hsp70, Hsc70, Hsp90, Bag-3 and Hsp110). As expected, we confirmed loss of DNAJA1 and interestingly did not observe any compensatory effects on the levels of the other chaperones/co-chaperones studied (Fig. S1). According to pharmacologic action, the compounds in the library have been divided into seven categories: protein synthesis inhibitors, proteasome inhibitors, epigenetic modifiers, metabolic inhibitors, cytoskeletal inhibitors, signal transduction inhibitors and DNA synthesis/repair inhibitors. Further fold enrichment of each drug category was calculated for the drugs whose potency increased or decreased with loss of DNAJA1. To monitor the screening quality, each screening plate contained control wells treated with vehicle (1% DMSO). The final concentration of the screening compounds was 50 μmol/L. Positive hits (synergistic) or negative hits (antagonistic) were determined by normalizing the log_2_ ratio of viability of *DNAJA1* knockout cells over wildtype cells. A full list of the screening results is shown in Supplementary Table T1 and the sorted data are graphically plotted in Fig. 2B. The effectiveness of a large proportion of anticancer molecules in the collection were impacted*,* with 41 of (31%) showing increased potency and 18 (14%) showing reduced potency upon loss of *DNAJA1* (Fig. 2C). Drug target analysis was carried out by calculating fold enrichment of positive hits (synergistic) or negative hits (antagonistic) over the total number of drugs in that category. Drug target analysis of the synergistic drug hits revealed significant enrichment in DNA synthesis and repair inhibitors, signal transduction inhibitors as well as cytoskeletal inhibitors (Fig. 2D). In contrast, drug target analysis of antagonistic drug hits revealed a higher enrichment in categories such as epigenetic modifiers, protein synthesis inhibitors, cytoskeletal inhibitors and proteasome inhibitors (Fig. 2E). For a full list of drugs in each category and raw data from screen, please see supplemental Table T1.

Strikingly, a small number of compounds with supposedly related function showed dissimilar alteration of potency upon loss of DNAJA1 function, potentially caused by off-target drug effects (see discussion).

### Validation of anticancer drugs significantly altered for potency upon loss of DNAJA1

**Figure 3:**
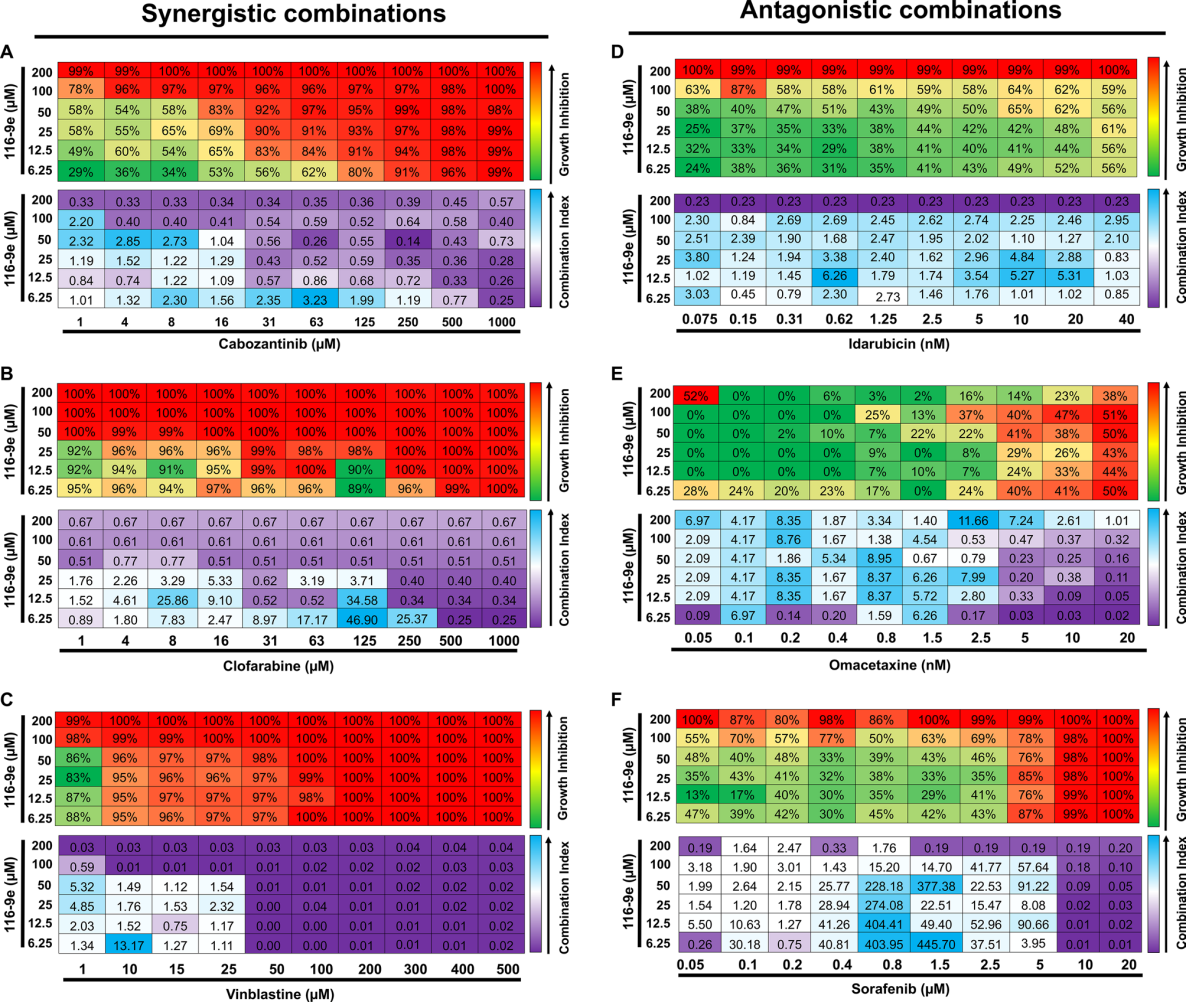
Drug interaction between 116-9e (DNAJA1 inhibitor) and selected hits. LNCaP cells were treated with different concentrations of cabozantinib (**A**), clofarabine (**B**), vinblastine (**C**), idarubicin (**D**), omacetaxine (**E**) and sorafenib (**F**) with or without 116-9e for 72 h in RPMI-1640 medium containing 10% FBS. Each point is the mean ± SD for three independent experiments. Growth inhibition was determined using Cell Titer-Glo assay. Combination Index (CI, measure of drug synergy) was determined using Chou-Talalay method via Compusyn software. CI values are as follows: < 0.1 (very strongly synergistic), 0.1–0.3 (strongly synergistic), < 0.9 (synergistic), 0.9–1.1 (additive), 1.1–3.3 (antagonistic), 3.3–10 (strongly antagonistic), > 10 (very strongly antagonistic).

Many anticancer compounds have low potency, poor therapeutic index or suffer from the development of resistance . Monotherapy is rarely efficient and instead drug cocktails are widely used in the clinic^16^. Establishing these combinations can enhance the scope of preclinical studies and inform the design of future clinical trials. Although knockout of *DNAJA1* substantially increased the potency of a number of anticancer molecules, it remained to be determined whether small-molecule inhibition of DNAJA1 could produce a similar result. Our previous bioinformatics analysis indicated that a large proportion of prostate cancer cells contain either amplification or mutation of *DNAJA1* (approximately 18%, see Fig. 1). To validate the results of our initial screen, we analyzed the effect of treating prostate cancer cells (LNCaP) with a combination of 116-9e, a small molecule inhibitor of DNAJA1^18^ and selected hits from our screen. We decided to focus on three synergistic drugs discovered in the screen: cabozantinib (receptor tyrosine kinase inhibitor), clofarabine (an RNR inhibitor)^19^ and vinblastine (microtubule inhibitor/G2 arresting agent)^20,21^. We also validated three drugs that demonstrated a significant loss of potency in cells lacking *DNAJA1*: sorafenib (a VEGFR-2 inhibitor)^22^, omacetaxine mepesuccinate (more commonly known as homoharringtonine, a protein translation inhibitor)^23^ and idarubicin (topoisomerase II inhibitor)^24^. To determine synergy in a quantitative manner, we calculated drug synergy (Combination Index values, CI) between 116-9e and either synergistic or antagonistic drugs hits across a broad range of concentrations using the Chou-Talalay method^25^ (for effects of individual drugs, please see Fig. S2). For three hits identified in our screen (cabozantinib, clofarabine and vinblastine) we confirmed significant synergy (CI < 1) with 116-9e across a range of doses (Fig. 3A–C). In contrast, idarubicin, omacetaxine and sorafenib displayed a significantly antagonistic interaction (CI > 1) across a range of doses (Fig. 3D–F). These data suggest that while DNAJA1 inhibition is a promising strategy to sensitize cells to a number of currently used anticancer drugs, the loss of DNAJA1 can significantly decrease the potency of a small subset of inhibitors.

### Evaluating the effects of dual targeting of identified drugs with DNAJA1 inhibition on morphology and viability of prostate cancer spheroids

**Figure 4:**
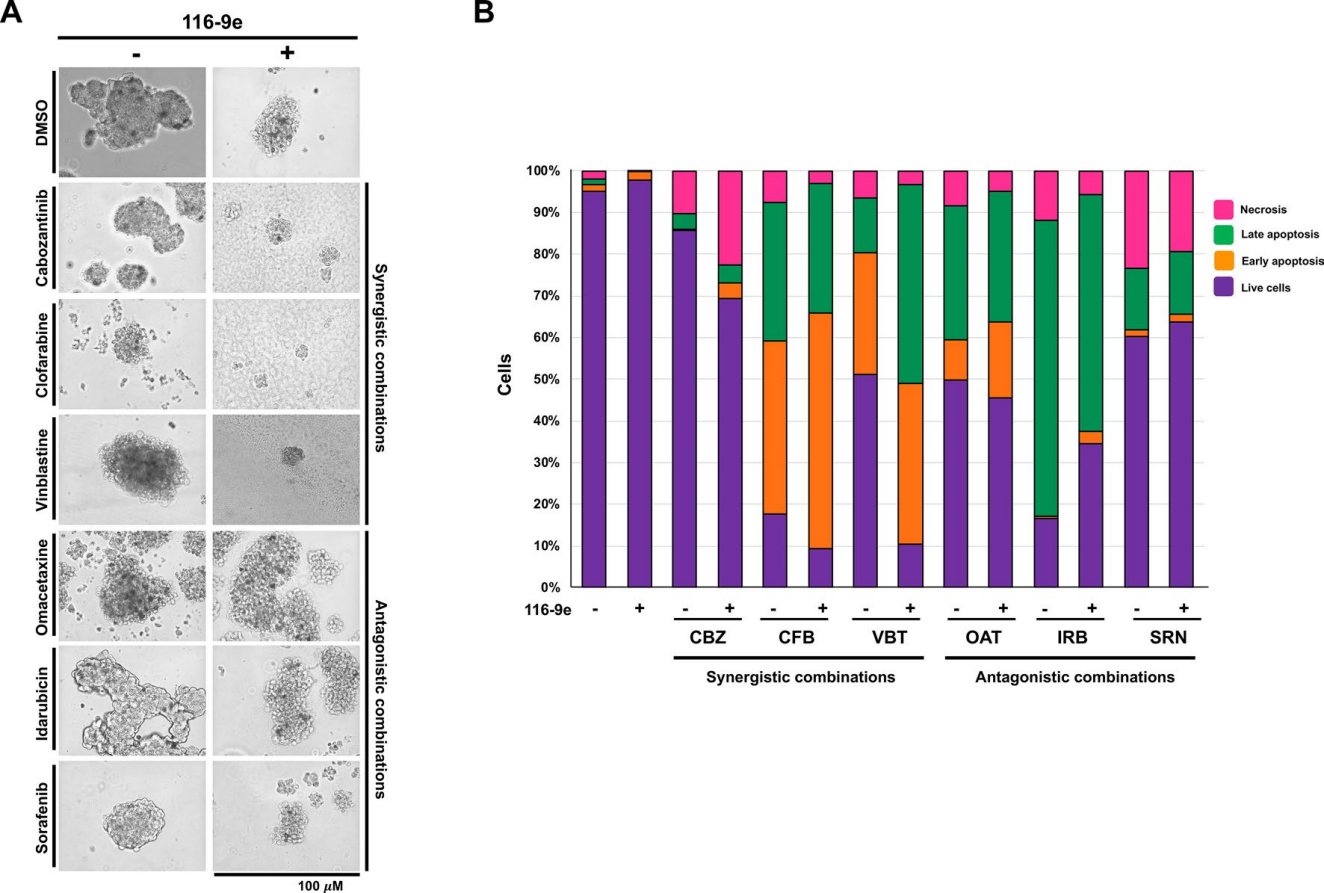
Effect of combination treatments on prostate cancer spheroids. (**A**) Cells were plated on Matrigel-coated 24 well plates. Six drugs (cabozantinib, clofarabine, vinblastine, idarubicin, omacetaxine and sorafenib) were tested on prostate cancer spheroids. These experiments were performed in triplicate and are average of 3 replicates from 3 different wells of a cell culture plate. The pictures are representative images as acquired using an EVOS cell imager. (**B)** Proliferation of spheroids treated with cabozantinib (CBZ), clofarabine (CFB), vinblastine (VBT), idarubicin (IRB), omacetaxine (OAT) and sorafenib (SRN) measured using AnnexinV/PI staining.

Recent studies have suggested that precision therapy approaches involving the exposure of drugs directly to the primary tumor tissue have the potential to augment the personalized medicine efforts and influence clinical decisions^26^. Establishing ex vivo three-dimensional (3D) tumor spheroids or organoids derived from primary cancers can be easily established and potentially scaled to screen drug combinations. These 3D cancer models appear to recapitulate features of the tumor of origin in terms of heterogeneity, cell differentiation, histoarchitecture, and clinical drug response and can be used for rapid drug screening^27^. We therefore next examined the effect of drug combination (three antagonistic and synergistic hits) on LNCaP spheroids. Specifically, changes in spheroid size and shape induced by the 3 antagonistic and synergistic drugs were determined. Visual examination revealed that for the synergistic drugs combination with 116-9e resulted in physical disruption of LNCaP spheroids, resulting in decrease in spheroid size (Fig. 4A). The disruption started on the second day of the treatment. However, when the 3 antagonistic drugs were administered along with 116-9e, there were minimal changes in spheroid morphology indicating that the combination was ineffective.

Next, we measured the induction of apoptosis in the spheroids post drug treatments. We determined the kinetics of apoptosis induction using AnnexinV/PI staining. Drug-induced apoptosis was readily detected in the LNCaP spheroids treated with mono and dual drug combinations. In concurrence with the previous results, the combination of the three synergistic drugs with 116-9e displayed enhanced apoptosis as compared to the single drug treatment whereas spheroids treated with the 3 antagonistic drugs showed little or no difference in the rate of apoptosis as compared to the dual drug combination with 116-9e (Fig. 4B).

## Discussion

Although inhibitors of Hsp70 and Hsp90 have been developed for research purposes, the conversion of these molecules for use in patient treatment have been hampered by toxicity issues^4^. We undertook this study to resolve conflicting literature on whether inhibiting DNAJA1, a co-chaperone of Hsp70 may be useful as a novel anticancer strategy. Our bioinformatic analysis of *DNAJA1* expression and mutation clearly identify *DNAJA1* as being highly altered in a range of cancers, particularly in prostate cancer. Interestingly, *DNAJA1* despite being substantially overexpressed in a range of cancers, there was minimal correlation between *DNAJA1* copy number and level of expression. While beyond the scope of this study, it is possible that the high levels of *DNAJA1* expression observed may be a result of increased transcription brought on hyperactive signaling pathways common in cancer cells. This data in conjunction with a recent finding that Hsp40 is involved in regulation of ARv^13^ and p53^28^ makes DNAJA1 inhibition an ideal choice as a novel therapeutic target in Prostate Cancer.

In this study, loss of DNAJA1 increased the potency of a substantial number (31%) of clinically used anticancer drugs. This increased potency may be related to the destabilization of clients that are the target of these small molecules. For example, Hsp70 activates many proteins involved in the DNA damage response and DNA repair pathways (DDR), including ATM, APE1, PARP1, XRCC1^29^. Recently, studies from our group have established roles for both Hsp70 and DNAJA1 in stability of the RNR complex^8,29,30^. It is unsurprising then that many of the anticancer agents displaying synergy with loss of DNAJA1 are connected to inhibition of the DNA damage response/repair. These include molecules such as 5-fluorouracil (5-FU), premetrexed, clofarabine, olaparib and niraparib etoposide, teniposide and valrubicin. Here we validated synergy with the RNR inhibitor clofarabine. Clofarabine is phosphorylated intracellularly to form cytotoxic active 5′-triphosphate metabolite, which inhibits the enzymatic activities of RNR and DNA polymerase, resulting in inhibition of DNA synthesis and repair^31^. While most DDR inhibitors displayed increased potency with DNAJA1 depletion, four of them were antagonistic to loss of DNAJA1. These include topoisomerase inhibitors and nucleic acid synthesis inhibitors such as trifluridine, irinotecan, epirubicin (4′-epi-isomer of the antibiotic doxorubicin) and idarubicin (4-demethoxy analogue of daunorubicin)^32^. While at first these results seem paradoxical, it is worth noting that inrinotecan is a type I topoisomerase inhibitor, whereas Etoposide (synergistic with loss of DNAJA1) is a type II topoisomerase inhibitor. It may be that Hsp70 and DNAJA1 play opposing regulatory roles in the stabilization and activation of these related proteins.

In addition to DDR, DNAJA1 is also involved in signal transduction, with previous reports indicating that the yeast homolog of DNAJA1 (Ydj1) is critical for supporting the integrity of kinase signaling networks^33^. DNAJA1 is mobilized to specific sites within the nucleus in response to inappropriate targeting or folding of specific mutant receptors. DNAJA1 overexpression ameliorates the defective transactivation and trans-repression activity of mutant Glucocorticoid receptors^34^. In line with the previous studies, we found that a handful of Receptor Tyrosine kinase inhibitors were synergistic with DNAJA1 depletion. These included Vascular endothelial growth factor receptor (VEGFR) inhibitors such as sunitinib, cabozantinib, lenvatinib and pazopanib. Interestingly, randomized phase III clinical trials are being conducted to validate the efficacy of Cabozantinib in heavily pretreated prostate cancer patients^35^. One implication from our study is that DNAJA1 inhibition might significantly enhance the effect of cabozantinib monotherapy.

Strikingly, some of the kinase inhibitors were antagonistic to DNAJA1 depletion. These include VEGFR inhibitors such as regorafenib and sorafenib. This disparity can be explained by the different target receptors and mechanisms of action of these drugs. Interestingly, recent studies indicated that these small molecule inhibitors exhibit off-target effects. Some of these drugs are misidentified and mischaracterized for their target specific inhibition, which has contributed to the high failure rate of these drugs in the treatment of cancer patients^36^.

In addition to its role in signal transduction, DNAJA1 is also important for maintaining the cellular cytoskeleton. Previous studies have suggested that YDJ1 (the yeast homolog of DNAJA1) is important for the proper assembly of microtubules^37^. Another report showed that DNAJA1 depletion causes relocation of N-cadherin and enhanced activity of metalloproteinases. This leads to changes in the actin cytoskeleton indicating that DNAJA1 is important for prevention of the amoeboid-like transition of tumor cells^38^. These studies indicated the involvement of DNAJA1 in maintaining cytoskeletal organization. We found 3 anticancer drugs targeting the cytoskeleton to be synergistic with DNAJA1 depletion, including vinblastine sulfate (cytoskeletal inhibitor that disrupts microtubule formation during mitosis and interferes with glutamic acid metabolism), estramustine (binds to microtubule-associated proteins (MAPs) and inhibits microtubule dynamics) and ixabepilone (promotes tubulin polymerization and microtubule stabilization, thereby arresting cells in the G2-M phase^39^. Strikingly, two of the tubulin inhibitors were found to be *antagonistic* to DNAJA1 depletion. These include paclitaxel and ixabepilone. Paclitaxel inhibits the disassembly of microtubules resulting in the inhibition of cell division whereas Ixabepilone promotes tubulin polymerization and microtubule stabilization, arresting cells in the G2-M phase of the cell cycle^39^. This apparent discrepancy may be explained by off-target effects of these molecules (see below).

Epigenetic modifying drugs display substantially modified potency depending on cellular DNAJA1 status. While previous studies have indicated the association between proteomic changes and histone PTMs in response to Hsp90 inhibitor treatment in bladder carcinoma cells, no such association has been shown for DNAJA1 and Histone PTMs^40^. Interestingly, vorinostat was the only drug that was synergistic to DNAJA1 inhibition. It is a histone deacetylase inhibitor that binds to the catalytic domain of the histone deacetylases (HDACs). However, we also identified two histone deacetylase inhibitor drugs to be antagonistic to DNAJA1 depletion**,** panobinostat and romidepsin. These inhibit histone deacetylase (HDAC) which may impact cell cycle protein expression, cell cycle arrest in the G2/M phase and apoptosis^41^. Excitingly, our data suggest a functional link between histones, their modifications and DNAJA1. While these findings require further investigation, it is possible that DNAJA1 may regulate the stability of histones themselves or histone chaperones. Interestingly, bortezomib (a proteasome inhibitor) lost potency when DNAJA1 was either inhibited with 116-9e or knocked out with CRISPR. Interestingly, a similar phenomenon has been observed in B16F10 melanoma cells. While treatment of these cells with 10 nM bortezomib was cytotoxic, this effect was not observed in cells treated with a combination of both quercetin (an Hsp70 inhibitor) and bortezomib^42^. This apparent antagonism may be explained by their mechanism of action on the heat shock transcription factor, HSF1. While bortezomib acts to trigger the heat shock response in some cancers, the Hsp70/co-chaperone system maintains HSF1 in an less active immature form^43–46^. It is interesting to note that while there are clear classes of drugs that are made more potent by loss of DNAJA1 function (DNA damage response, cytoskeletal function etc.), there are a small number of drugs in these classes that are not impacted at all or even made less potent. This apparent discrepancy implies that some of these inhibitors might have multiple cellular targets in addition to their proposed primary mechanism of action. This theory has been validated in fascinating studies comparing effects of small molecule therapies, gene knockout and knockdowns that theoretically target the same genes^36^.

As in the case of any chemogenomic screen, care must be taken to validate screening results with other methods. While CRISPR KO cell lines offer the advantage of complete, specific and permanent gene silencing as compared to transient inhibition seen with small molecules or siRNA, the authors acknowledge that permanent knockout lines may respond by overpexressing/suppressing other genes to compensate, leading to non-specific effects. In this study, we took the approach of following up our intitial screen with small molecule validation in 2D and 3D cell culture models. Going forward, we intend to validate several of these hits in vivo (mouse) model systems. 116-9e is a relatively new and unexplored molecule; while it clearly alters JDP binding, the exact impact on all Hsp70-JDPs has not been characterized^8,18^. Interestingly, the *DNAJA1* knockout cell line grows effectively the same as WT and our examination of key chaperone protein levels do note reveal any major alterations. However, we do acknowledge that it is possible that the *DNAJA1* knockout cell line may have adapted by overexpressing other JDPs such as DNAJA2, DNAJB1 or DNAJB6. In future studies, we hope to investigate this further by quantitating the proteome in HAP1 cells (WT and *DNAJA1* knockout) in both untreated and 116-9e treated conditions. Given the essential nature of Hsp70/Hsc70 in cancer cells, if 116-9e truly inhibited all JDP interactions it would be highly toxic to cells which we do not observe, suggesting there must be some selectivity in JDP inhibition.

Recent studies from our group and other have described the clear impact of Hsp70/JDP inhibition on individual oncoprotein client stability and prostate cancer cell survival^8,13^. Overall, this study demonstrates the larger feasibility of inhibiting Hsp70 co-chaperones such as DNAJA1 as a novel anticancer therapy, acting to fine-tune Hsp70 function rather than completely abolishing it. Nearly a third of the anticancer compounds screened demonstrated increased potency in *DNAJA1* knockout cells. Rather than attempting to develop co-chaperone inhibitors as a monotherapy, we believe their strength lies as sensitizing agents to existing therapies. Moreover, our data imply that overexpression of *DNAJA1* in patient tumors may impact the effectiveness of a number of commonly used anticancer drugs. While further experiments characterizing (1) the specific DNAJA1-mediated effects in cancer proliferation and (2) the specificity of drugs such as 116-9e are required, our studies suggest perhaps a future precision medicine approach that uses tumor *DNAJA1* status to guide treatment strategy.

## Materials and methods

### Cell culture

The HAP1 Chronic Myelogenous Leukemia cancer cell line and *DNAJA1* knockout cell line was purchased from Horizon Discovery and were cultured in Iscove’s Modified Eagle Medium (Invitrogen) with 10% fetal bovine serum (Gibco), 100 units/ml penicillin, and 100 μg/ml streptomycin at 5% CO_2_ and 37° C. The LNCaP cancer cell line was purchased from ATCC and were cultured in RPMI-1640 medium (Invitrogen) with 10% fetal bovine serum (FBS, Clontech), 100 units/ml penicillin, and 100 μg/ml streptomycin at 5% CO_2_ and 37° C.

### Drug screening

Approved Oncology Drug plates consisting of the most current FDA approved anticancer drugs were obtained from the National Cancer Institute (NCI). For experiments delineating the synergy between the loss of DNAJA1 and approved anticancer drug, HAP1 cells and HAP1 (*DNAJA1* KO) cells were plated in growth media at 20% confluency 1 day prior to drug treatment. On Day 1 of treatment, cells were treated with DMSO (control), Approved oncology anticancer drugs at 50 µM for 72 h. Following drug treatments, Cell Titer-Glo reagent was added directly to the wells according to the manufacturer’s instructions. The luminescence was measured on Bio-Tek Plate reader. Luminescence reading was normalized to and expressed as a relative percentage of the plate averaged DMSO control. The data shown are the mean and SEM of three independent biological replicates.

### Combination index (CI) calculations

For IC_50_ calculations, LNCaP cells were seeded in triplicates in 96-well white bottom Nunc plates in growth media at 20% confluency 1 day prior to initiation of drug treatment. On Day 1 of treatment, cells were treated with DMSO (control) and ten folds serial dilution of anti-cancer drugs cabozantinib, clofarabine, vinblastine, sorafenib, idarubicin and omacetaxine mepesuccinate and 116-9e. After 72 h, cell viability was measured using Promega Cell Titer-Glo cell viability assay on Bio-Tek plate reader. The combination index was calculated using the Chou-Talalay method using CompuSyn software^47^.

### Spheroid generation

Single-cell suspensions (5,000/well) were plated in one well of 24-well plates in a 1:1 mixture of RPMI medium and Matrigel (BD Bioscience CB-40324). Cells in Matrigel were kept cold at all times and under continuous agitation. Warm PBS was added to all empty wells, if any. Plates were incubated at 37 °C with 5% CO_2_ for 15 min to solidify the gel before addition of 100 µl of pre-warmed RPMI to each well. Two days after seeding, the media was fully aspirated and replaced with fresh RPMI containing the indicated drugs. The same procedure was repeated daily on two consecutive days. Twenty-four hours after the last treatments, the media was aspirated and the wells were washed with 100 µl of pre-warmed PBS. To prepare for downstream assays, spheroids were released from the Matrigel by incubating at 37 °C for 40 min in 100 µl of 10 mg/mL Dispase (Sigma).

### Apoptosis assay

Apoptosis of LNCaP spheroids was detected by the Annexin V–FITC/propidium iodide–binding assay. Cells were treated with either 0.1% DMSO (dimethyl sulfoxide),116-9e, cabozantinib, clofarabine, vinblastine, sorafenib, idarubicin, omacetaxine mepesuccinate and sorafenib alone or in combination with 116-9e for 48 h at the IC_50_ concentrations, and then stained with Annexin V–FITC and propidium iodide. The rate of apoptosis was determined using a BD LSR Fortessa flow cytometer, and the collected data were analyzed using FlowJo software. Apoptosis was reported as the mean ± SD. The results are representative of three independent experiments.

### Bioinformatics

Cancer genome data and Cancer Cell Line Encyclopedia data were accessed from the cBioPortal (www.cbioportal.org) for Cancer Genomics ^48^. Total patient numbers and detailed information regarding published datasets and associated publications are indicated in Fig. 1A,B.

### Statistical analysis

Data were analyzed using GraphPad Prism built-in statistical tests indicated in relevant figure legends. The following asterisk system for P-value was used: P < 0.05; P < 0.01; 0.001; and P < 0.0001.

### Western blotting

Protein extracts were made as described^8^. 30 μg of protein was separated by 4–12% NuPAGE SDS-PAGE (Thermo). Proteins were detected using the following antibodies; anti-DNAJA1/HDJ2 (Thermo # MA5-12748), anti-Actin (CST # 9774), Anti-Hsc70 (Santa Cruz, # sc-7298), anti-Hsp70 (Enzo # C92F3A­5), anti-Hsp90 ⍺/ℬ (Santa Cruz # sc-13119), anti-Bag3 (Santa Cruz # sc-136467), anti-Hsp110 (Stress Marq, # SPC-195),) at 1:4,000 dilution in TBST + 1% BSA. The secondary antibody (StarBright Blue 700 Fluorescent Secondary Mouse) was used at 1:3,000 dilution in TBST + 1% BSA. Blots were imaged on a Chemi Doc MP imaging system (Bio-Rad).

## Supplementary information

Supplementary Figures

Supplementary Table T1
