# Supplementary material for: Chemogenomic screening identifies the Hsp70 co-chaperone DNAJA1 as a hub for anticancer drug resistance
Source: Sci Rep. 2020 Aug 14;10:13831. doi: 10.1038/s41598-020-70764-x (PMC7429498; doi:10.1038/s41598-020-70764-x)
Supplement: Supplementary file 1 — Supplementary Figures [file 41598_2020_70764_MOESM1_ESM.pdf]

Supplementary information for:

Chemogenomic screening identifies the Hsp70 co-chaperone DNAJA1 as a hub for anticancer drug resistance

Nitika[1], Jacob S. Blackman[1], Laura E. Knighton[1], Jade E. Takakuwa[1], Stuart K. Calderwood[2] and Andrew W. Truman[1]*

[1]Department of Biological Sciences, University of North Carolina Charlotte, Charlotte, NC 28223, USA
[2]Department of Radiation Oncology, Beth Israel Deaconess Medical Center, Harvard Medical School, 330 Brookline Ave, Boston, MA 02215, USA

*Corresponding author
E-mail: A.Truman@uncc.edu

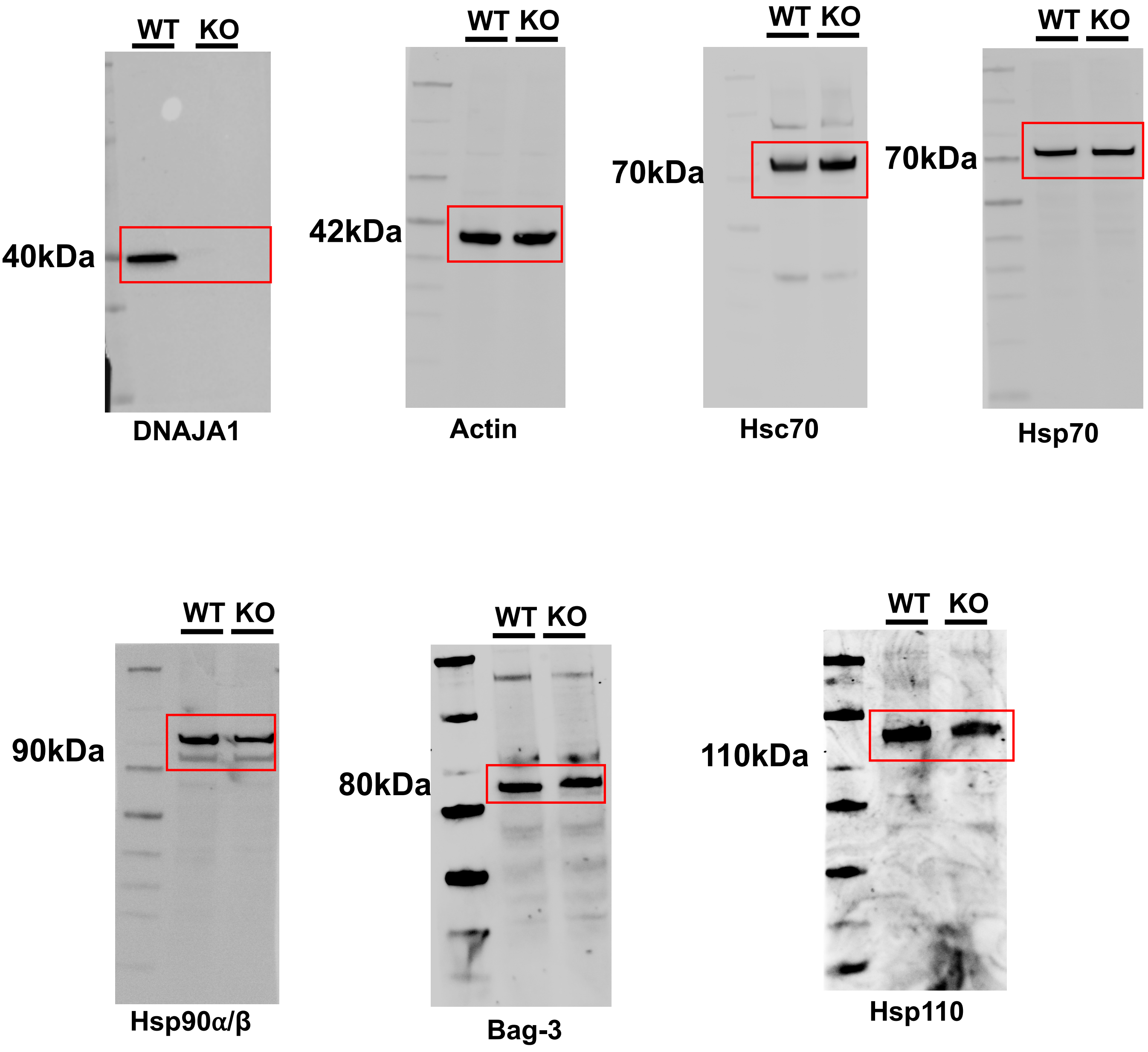

Fig. S1. Expression of various chaperone/co-chaperone proteins in HAP1 WT/DNAJA1 knockout cells. Cell lysates extracted from HAP1 WT and DNAJA1 CRISPR KO cells were resolved on SDS-PAGE gels and further processed by immunoblotting with anti-DNAJA1, Actin, Hsc70, Hsp70, Hsp90, Bag-3, Hsp110 and Actin antibodies.

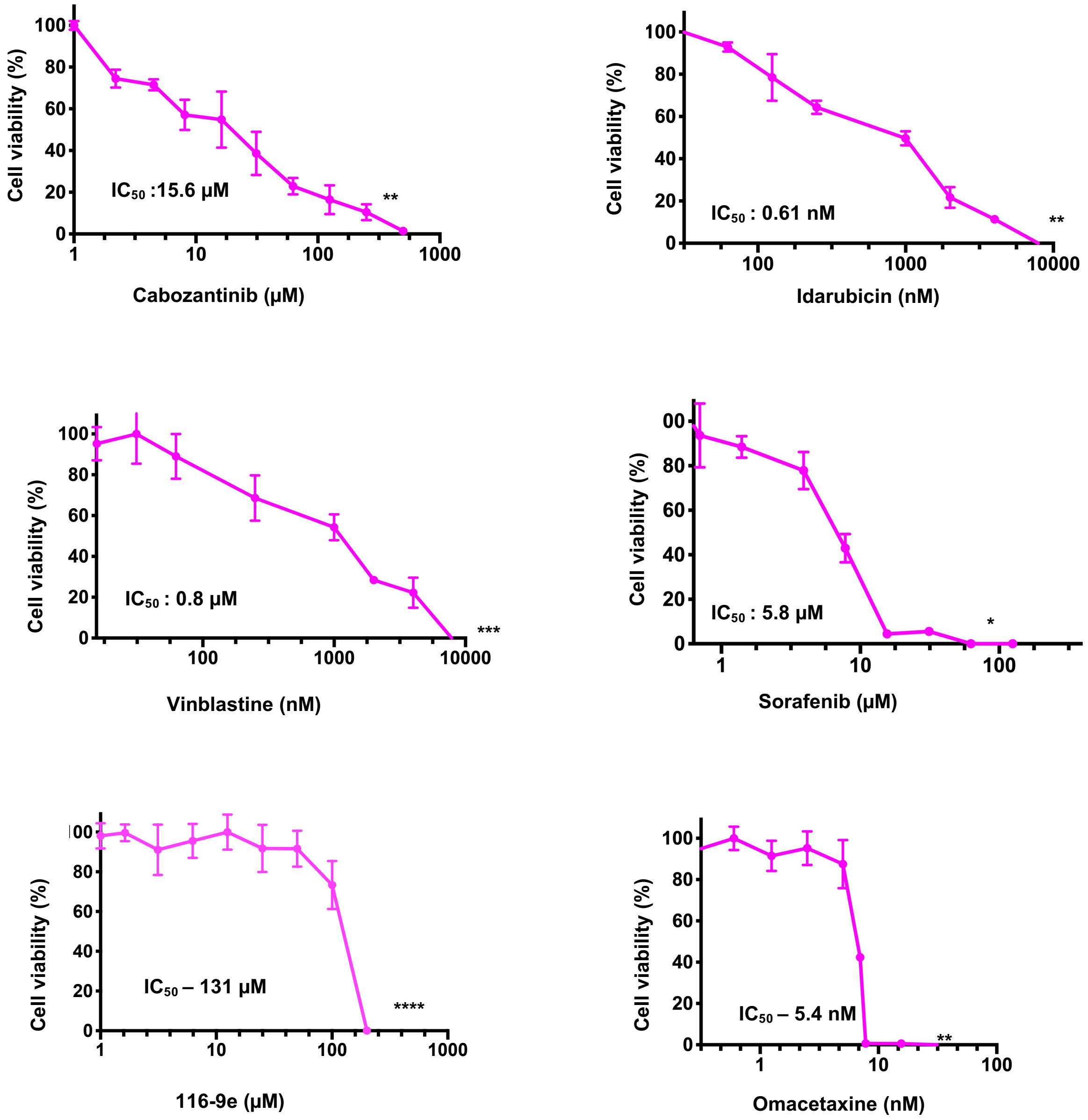

Fig. S2. Effects of selected FDA-approved drugs on LnCaP cells. LNCaP cells were treated with serial dilutions of indicated drugs for 3 days. Cell viability was determined using Cell Titer Glo assay and results shown are average and SD from three replicates (***P<0.001 compared to DMSO control, t-test).
